# A tissue-specific promoter derived from a SINE retrotransposon drives biallelic expression of *PLAGL1* in human lymphocytes

**DOI:** 10.1371/journal.pone.0185678

**Published:** 2017-09-28

**Authors:** Claire E. L. Smith, Alexia Alexandraki, Sarah F. Cordery, Rekha Parmar, David T. Bonthron, Elizabeth M. A. Valleley

**Affiliations:** School of Medicine, University of Leeds, St. James’s University Hospital, Leeds, United Kingdom; International University of Health and Welfare School of Medicine, JAPAN

## Abstract

The imprinted gene *PLAGL1* is an important regulator of apoptosis and cell cycle arrest. Loss of its expression has been implicated in tumorigenesis in a range of different cancers, and overexpression during fetal development causes transient neonatal diabetes mellitus (TNDM). *PLAGL1* lies within an imprinted region of chromosome 6q24, and monoallelic expression from the major, differentially methylated promoter (P1) occurs in most human tissues. However, in peripheral blood leukocytes, the active promoter (P2) is non-imprinted and drives biallelic transcription. We report here a novel *PLAGL1* promoter (P5) derived from the insertion of a primate-specific, MIR3 SINE retrotransposon. P5 is highly utilized in lymphocytes, particularly in T cells, and like P2, directs biallelic transcription. Our results show that it is important to consider P5 in relation to *PLAGL1* function in T cells when investigating the dysregulation of this gene.

## Introduction

Genomic imprinting is an epigenetic process by which specific genes are expressed preferentially according to their parent of origin. *PLAGL1* (also known as *ZAC*, *LOT1* and *Zac1* in mouse) is an imprinted gene that is a key regulator of a network of other imprinted genes, involved in embryonic growth and development [1]. At a biochemical and cellular level, PLAGL1 protein both acts as a transcriptional co-activator for p53 and regulates cell cycle and apoptosis concomitantly [2,3]. Dysregulation of this gene plays a pathogenic role in the tumorigenesis of several types of cancer and in a rare form of childhood diabetes, transient neonatal diabetes mellitus (TNDM1; OMIM #601410) [4]. There is evidence that *PLAGL1* acts as a tumour suppressor in many tissues, as down-regulation has been observed in a range of different tumours, through hypermethylation of the imprinted promoter, chromosomal deletion or loss of heterozygosity [2,3]. Conversely, *PLAGL1* can also act as an oncogene in glioblastoma [5].

Most imprinted genes are located in clusters, across which there are some degrees of co-ordinate gene regulation; however, the *PLAGL1* locus on chromosome 6q24 has been shown to be a “micro-imprinted” domain [6]. It contains a differentially methylated region (DMR) that acts as a promoter (P1) directing transcription from the unmethylated, paternal allele in most human and mouse tissues [2]. Monoallelic expression occurs in most human fetal and adult tissues, with biallelic expression in peripheral blood leukocytes [4,7,8]. Over-expression of *PLAGL1* during fetal development, either secondary to paternal uniparental disomy of 6q24 or due to epigenetic alterations at the *PLAGL1* DMR, causes TNDM [2,4].

Previously, we defined and characterised a second promoter (P2) located within an unmethylated CpG island of human *PLAGL1*. Unlike P1, P2 drives biallelic transcription and is utilized predominantly in peripheral blood leukocytes [7]. Where detectable, P2 transcription is similarly biallelic in other human tissues [7]. We found that P2-derived *PLAGL1* expression is down-regulated in some cases of diffuse large B-cell lymphoma and the mechanism of the down-regulation did not involve hypermethylation of the P2 CpG island [9]. In addition, two minor, intragenic promoters have also been identified (P3 and P4), that like P1, produce paternally-expressed transcripts [6]. Although the biological drivers for the existence of multiple *PLAGL1* promoters are unclear, it appears that they may control tissue-specific expression or act as a protective mechanism to prevent loss of *PLAGL1* expression in some tissues. In this study, we have identified a fifth promoter region (P5), from which transcripts are highly expressed in lymphocytes, particularly T cells.

## Results and discussion

### *PLAGL1* transcripts are generated from a novel, fifth promoter

The present work was prompted by the existence of three novel spliced *PLAGL1* ESTs that appear to initiate at a novel genomic location lying between the differentially methylated (P1) promoter and the upstream, unmethylated promoter (P2). These ESTs range in length from 519-560-bp and were derived from peripheral blood mononuclear cells (accession number DA814732), thymus (DB104173) and kidney tumour tissue (DB177852) [10]. Sequence alignment indicated that they share a novel 5′ exon (>45-bp in size), that was neither annotated as part of a *PLAGL1* 5′-UTR in the UCSC genome browser, nor had been previously observed by us in transcripts isolated from several human tissues [7]. The DB177852 EST also contains a unique second exon, not observed in *PLAGL1* transcripts originating from P2. The remaining sequence in the EST transcripts aligned with known exons that constitute the 5′-UTR of *PLAGL1*; none of these ESTs extend as far as the *PLAGL1* coding region at their 3' ends. More recently, the sequence data from these ESTs have been combined in the UCSC genome browser as a curated transcript attributed to *PLAGL1*: NM_001317159.

### *PLAGL1* expression from the P5 promoter is highly expressed in T cells

**Fig 1:**
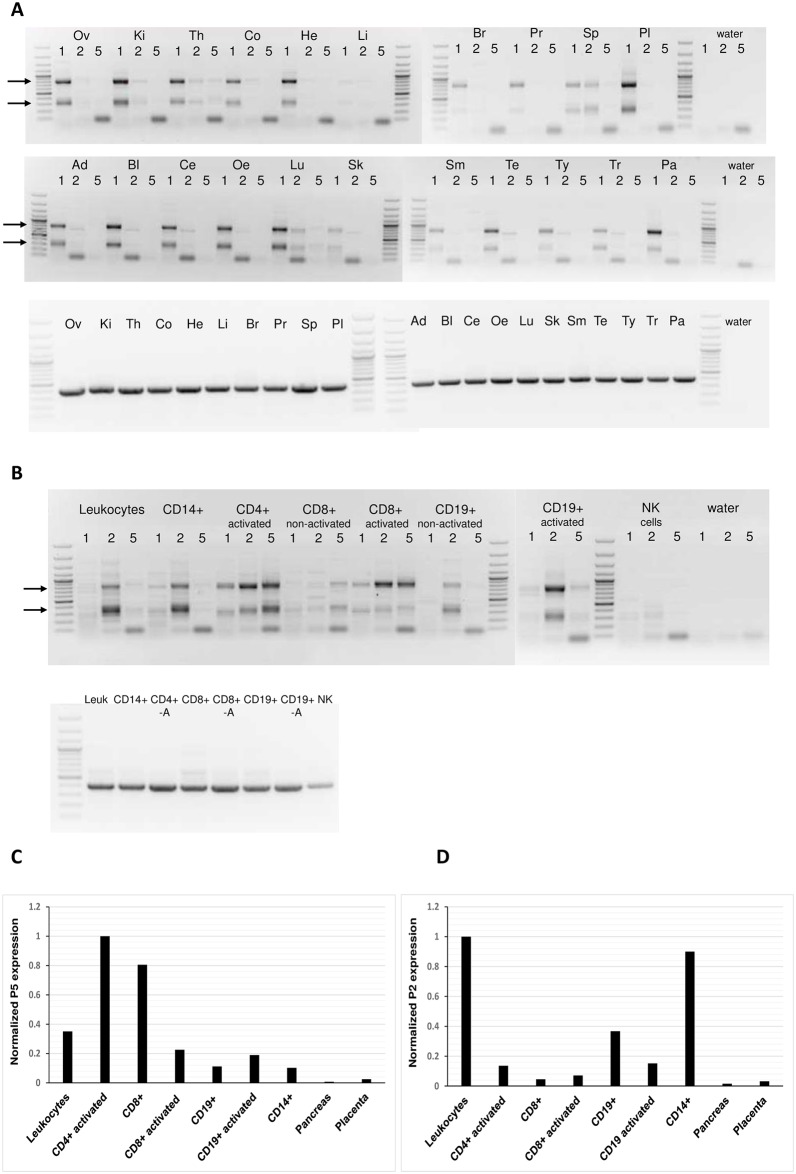
**Fig 1A: Comparison of *PLAGL1* transcription derived from three alternative promoters (P1; P2; P5) in a panel of human tissues**. For each tissue, three individual RT-PCR reactions were performed, using primers that specifically amplify transcripts from P1 (lane ‘1’), P2 (lane ‘2’) or P5 (lane ‘5’) (See Materials and methods). The top two panels show *PLAGL1* in cDNA samples: Ov, ovary; Ki, kidney; Th, thymus; Co, colon; He, heart; Li, liver; Br, brain; Pr, prostate; Sp, spleen; Pl, placenta; Ad, adipose tissue; Bl, bladder; Ce, cervix; Oe, oesophagus; Lu, lung; Sk, skeletal muscle; Sm, small intestine; Te, testes; Th, thyroid; Tr, trachea; Pa, pancreas. The molecular weight marker is GeneRuler 100bp+ ladder (Invitrogen). *PLAGL1* transcripts are subject to complex alternative splicing of the 5′-UTR, and the two major bands (arrowed) result from alternative splicing of the coding exons. The third panel down shows RT-PCR of the housekeeping gene *RPLP0* as a loading control for each reaction. All non-template (water) controls were negative. **Fig 1B: Comparison of *PLAGL1* transcription from the three alternative promoters in primary blood cells.** cDNA samples: leukocytes (peripheral blood leukocytes); CD14+ cells (monocytes); activated CD4+ cells (T helper/inducer cells); CD8+ cells (T suppressor/cytotoxic cells); activated CD8+ cells; CD19+ cells (B-lymphocytes); activated CD19+; NK cells (see Materials and methods). The panel below shows *RPLP0* as loading controls for each reaction. All non-template controls were negative. **Fig 1C: qPCR analysis of promoter P5 transcription in a range of blood cell RNAs, compared with pancreas and placenta tissue.** The expression levels of P5 transcripts were normalised to that of the endogenous control gene *RPLP0* in each sample, and are expressed in arbitrary units. **Fig 1D: qPCR analysis of promoter P2 transcription using the same cDNA samples as in Fig. 1C, for comparison.** Similar to the P5 qPCR, expression levels were normalised to RPLP0 expression, and are in arbitrary units.

We designate the putative new *PLAGL1* promoter from which these three ESTs derive as P5. We assessed its utilization by semi-quantitative RT-PCR, and compared it to P1 and P2 promoter activity in a panel of RNAs from a range of human tissues (Fig 1A). *PLAGL1* transcripts were amplified from each promoter separately, using a forward primer specific for either the P1, P2 or P5 5′-UTR exon, combined with a common reverse primer located in the coding region within the last exon. For each tissue, the PCR reactions were run in adjacent lanes on agarose gels for comparison with each other (Fig 1A). Specificity of transcripts derived from each promoter was confirmed by cloning and sequencing a sample of PCR products (data not shown).

Consistent with our previous study [7], we found that *PLAGL1* was expressed predominantly from the P1 promoter in most tissues, with a low level of P2 transcripts in some, such as spleen and lung (Fig 1A). The transcripts captured by the RT-PCR reaction vary in size due to complex alternative splicing of the 5'-UTR, and two main species are represented, that differ by ~475-bp due to inclusion or exclusion of the penultimate coding exon. These two mRNA classes encode the long and short PLAGL1 isoforms, with five zinc fingers present in the short isoform of the protein compared to seven zinc fingers in the long isoform [11]. P5 transcripts appeared to be rare compared to P1 or P2 transcripts, and were absent or barely detectable in all tissues, with only primer-dimer bands evident in the P5 lanes (Fig 1A). For P1 and P2 expression, the results are in general agreement with our previous observations in these tissues, as determined by qPCR [7].

As two of the deposited ESTs had been derived from cells of haemopoietic origin, we performed a similar analysis using an RNA panel from a range of blood cell types: peripheral blood leukocytes, CD14+, activated CD4+ and activated/non-activated samples of CD8+ and CD19+ cells (Fig 1B). RNA from non-activated CD4+ cells was not tested. In all samples tested (except CD8+ cells), *PLAGL1* was expressed more highly from the P2 promoter than P1, in agreement with our previous observations [7,9]. *PLAGL1* expression was only just detectable in NK cells. P2 promoter activity was predominant in peripheral blood leukocytes, CD14+ monocytes, and activated/non-activated CD19+ B cells. Expression from P5 was detectable in these cell types at a low level (Fig 1B) and primer-dimer bands were present, as in Fig 1A. However, in T cells (CD4+ and CD8+ samples) we observed particularly high expression from the P5 promoter. In all three samples (activated CD4+ cells; activated and non-activated CD8+ cells), this promoter was utilized at a higher or equivalent level to the blood cell-specific P2 promoter (Fig 1B).

To confirm the utilization of the P5 promoter in lymphocytes, we undertook qPCR using blood cell RNA samples and compared them with two other human tissues, pancreas and placenta, that express *PLAGL1* [7]. A custom-made Taqman assay was designed specifically to amplify P5 transcripts, and activated CD4+ cDNA was used to generate standard curves (due to its high expression level of P5) for the absolute quantification method. Expression of P5 was normalised to expression of the endogenous control gene, *RPLP0*. NK cells were not tested.

QPCR results showed highest expression of P5 transcripts in activated CD4+ cells and non-activated CD8+ cells, with lower levels in leukocytes and activated CD8+ cells (Fig 1C). P5 expression was observed in all blood cell types tested. P5 transcripts were not expressed in pancreas or placenta although high expression levels of *RPLP0* were observed, acting as a positive control. These qPCR data broadly agreed with our RT-PCR results, indicating that P5 transcripts are expressed predominantly in blood cells, particularly T cells. Some variability in expression levels between Fig 1B and 1C was observed: for example, P5 expression in activated CD8+ cells appeared lower by qPCR (Fig 1C) compared to RT-PCR (Fig 1B). This may be due to minor variation between RNA samples, as a second set of RNAs were used for qPCR compared to those previously used for RT-PCR, although samples were obtained from the same commercial source. Alternatively, there may be small differences in detection of alternatively-spliced P5 transcripts using the two techniques.

For comparison with Fig 1C, we used the same cDNAs to detect P2 transcripts, using a Taqman assay specific to P2 transcripts, as described previously [7] (Fig 1D). Lower levels of P2 expression in CD4+ activated cells necessitated using leukocyte cDNA for standard curves, therefore these data are presented on separate graphs. The highest levels of P2 transcripts were detected in leukocytes and CD14+ cells, with low levels in all of the blood cell samples tested. P2 expression was detected in all blood cell types tested but not in pancreas or placenta, confirming our previous observations [7]. P1 expression was not tested due to limited availability of RNA, although we would predict little or no P1 expression in blood cells and high levels in pancreas and placenta, based on our RT-PCR results (Fig 1A and 1B).

Overall, *PLAGL1* transcripts derived from the P5 promoter were found to be highly expressed in T cells, and in B cells to a lesser extent, compared to other blood cells or tissues. We also observed that transcripts encoding the long protein isoform were expressed predominantly in CD8+ cells (Fig 1B). This isoform, with seven zinc fingers, possesses a greater ability than the short form (referred to as PLAGL1Δ2) to induce apoptosis, suggesting that this may be a particular function of the protein in this cell type [11].

**Fig 2:**
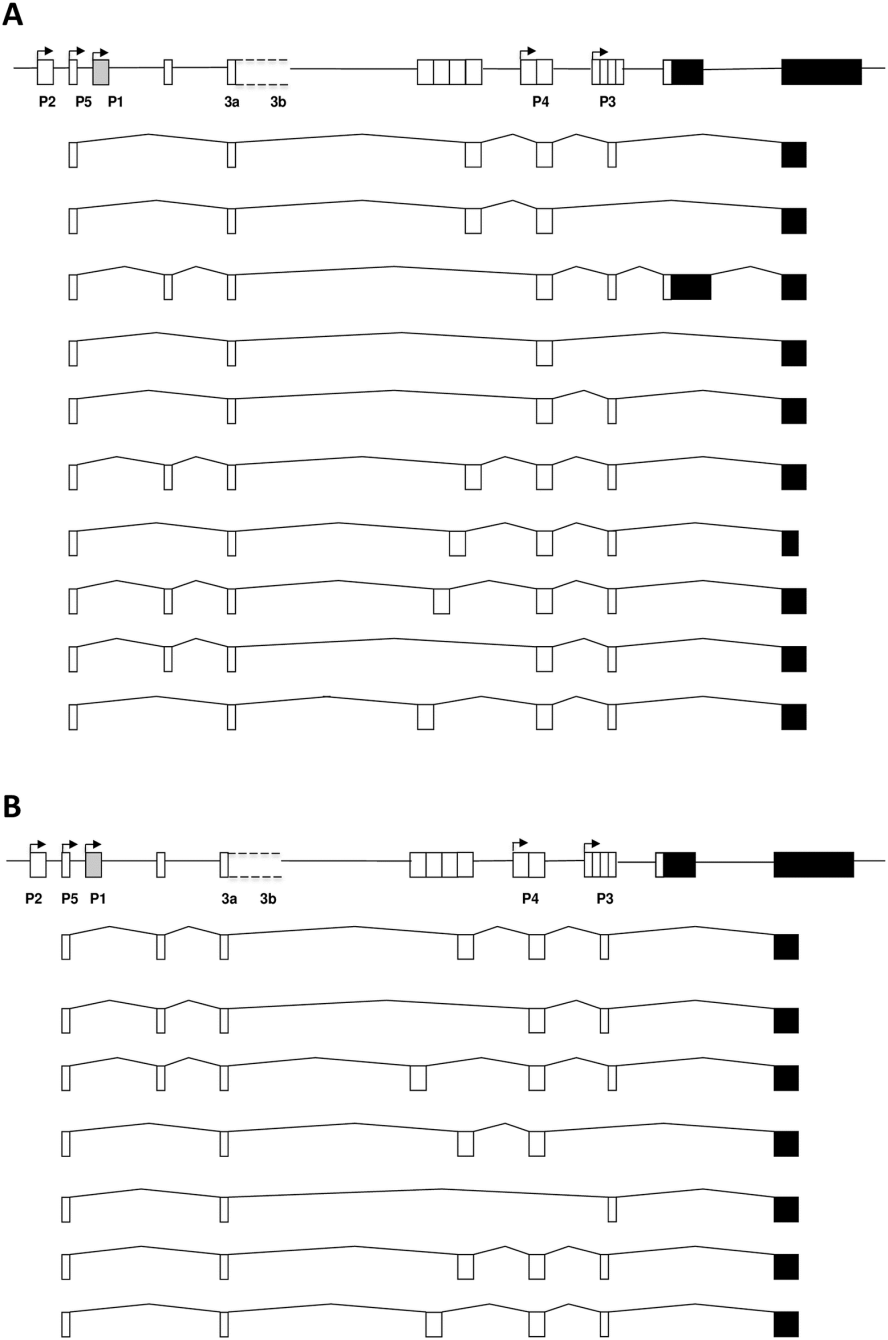
Alternative splicing of *PLAGL1* P5 transcripts isolated from A) activated CD4+ cell cDNA and B) activated CD19+ cell cDNA. The locus is shown at the top with the location of the five *PLAGL1* promoters indicated, and exon ‘3a’ and ‘3b’ (not to scale).

P5-derived RT-PCR products from activated CD4+ and activated CD19+ cell RNA were purified, cloned and sequenced to confirm that they were genuine *PLAGL1* transcripts. As for Fig 1B, transcripts were amplified using a forward primer in the P5 exon in combination with a reverse primer in the final coding exon. Sequencing confirmed that P5 transcripts extend to the *PLAGL1* coding region (Fig 2). Complex alternative splicing of the 5′-UTR was observed, and both the long (PLAGL1) and short (PLAGL1Δ2) protein isoforms were represented. Short protein isoforms predominated, which may be due to cloning bias of smaller transcripts. Our cloning strategy was not exhaustive and these transcripts may represent only a proportion of the alternatively spliced variants produced in these cell types.

### Confirmation of P5 transcriptional start sites (TSS) in B and T lymphocytes

To confirm that P5 represents a novel promoter region and does not splice to exons farther upstream within the *PLAGL1* 5′-UTR, we carried out 5′-RLM-RACE to determine the TSS for P5. As P5 expression is most abundant in lymphocytes, we used RNA derived from activated CD4+ cells (T lymphocytes) and activated CD19+ cells (B lymphocytes). The 5′-UTR of *PLAGL1* is subject to complex alternative splicing, but we have previously noted a ubiquitous 72-bp exon that appears to be obligatory (previously designated ‘exon 3a’) [7]. Therefore, RLM-RACE was carried out using nested reverse primers designed to this exon, combined with nested forward primers to the adaptor sequence (see Materials and methods).

**Fig 3:**
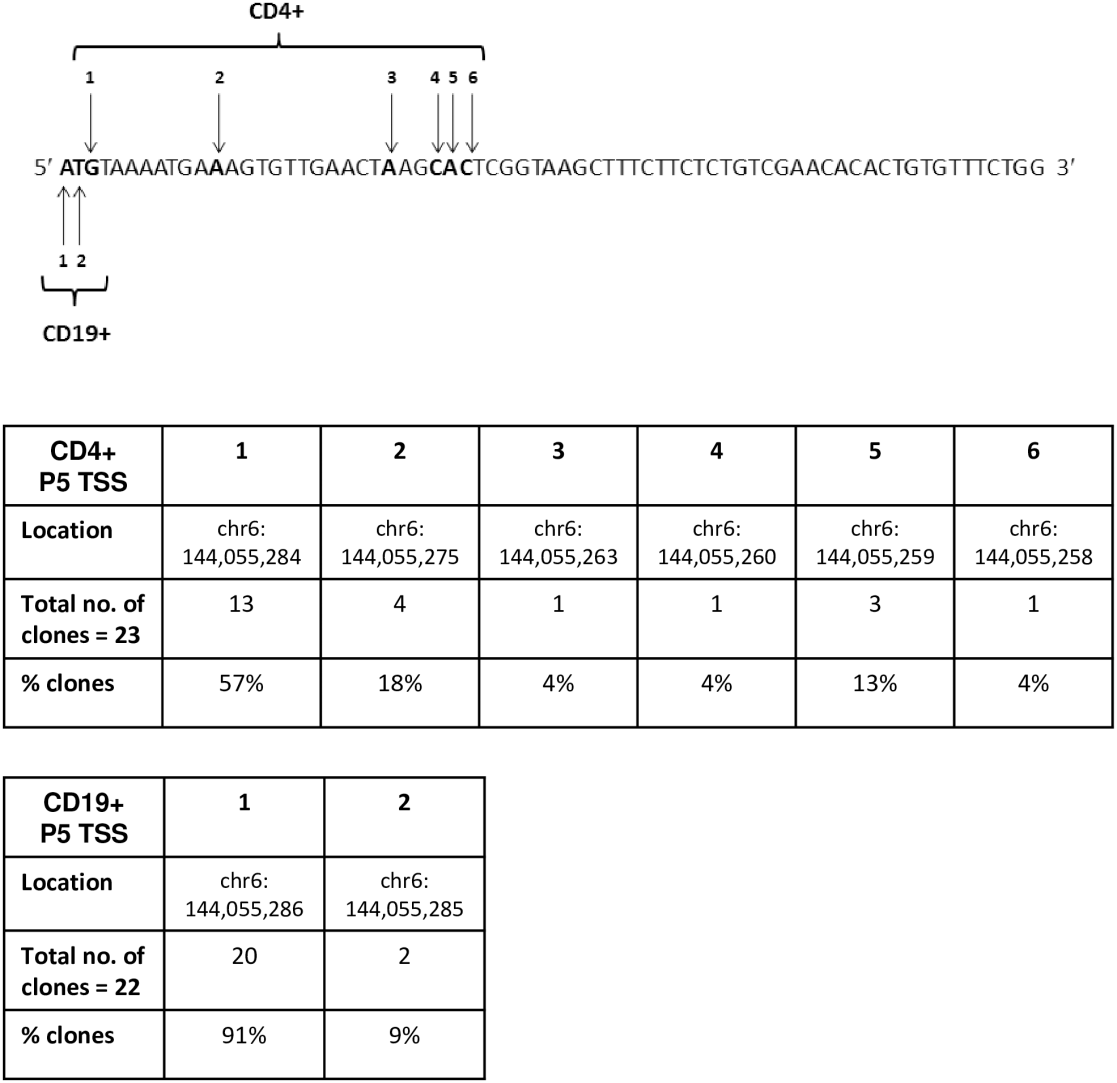
Transcriptional start sites identified for the *PLAGL1* P5 promoter in activated CD4+ and activated CD19+ cells. The complete P5 exon, as defined by 5′-RLM-RACE in these cell types, is 70-bp in length (chr6:144,055,217–144,055,286; genome assembly GRCh38/hg38). Arrows indicate the different transcriptional start sites identified in cloned 5'-RACE transcripts. The corresponding number in the Table indicates the number of clones corresponding to that TSS and their prevalence, expressed as a percentage of the number of clones sequenced.

Sequencing of the resulting PCR products showed the transcripts to be derived from the P2 or P5 promoters; since the adaptor primers were not specific for a particular promoter; this most likely reflects the predominance of P2- and P5-derived transcription in these cell types. We did not observe any P1 transcripts in the sample of clones sequenced, which may reflect the lower abundance of P1 transcripts. To determine the extent of the P5 exon, 45 cloned P5 5′-RACE transcripts were sequenced (Fig 3). The results confirmed that P5 is a genuine alternative promoter region and that the P5 exon is approximately 70-bp in size. The TSS for P5 transcripts in activated CD4+ and activated CD19+ cells differed by only a few base-pairs, but were distinct for each cell type (Fig 3). Potential ATG start codons are present in this 5′-UTR sequence, but they are not in frame with the coding sequence for *PLAGL1*.

### The P5 promoter region does not constitute a CpG island (CGI)

The P5 promoter lies ~9 kb downstream of the unmethylated P2 CGI and ~47 kb upstream of the differentially methylated P1 promoter (Fig 2). No CGI is identified in this genomic region by the UCSC Genome Browser. Using default parameters, MethPrimer (http://www.urogene.org/methprimer/index1.html) does not identify any CGIs either (defined as regions >100-bp, with a GC content >50% and an observed/expected CpG-rate of >0.6) within the 4-kb region surrounding the P5 start site. Similarly, analysis using *PROMO 3*.*0* (www.algen.lsi.upc.edu/recerca/menu_recerca.html) did not identify any CpG islands. However, ENCODE project data (visualized using the UCSC genome browser) show epigenetic features suggesting a promoter in this region. A DNAse I hypersensitivity site cluster overlaps the P5 exon and is reported in 17 cell lines, most of which are of haemopoietic origin. A histone H3K27Ac mark is also present, as an indicator of active regulatory elements [12].

Although no CGI is present, we used bisulphite sequencing to assess the methylation status of the CpG sites near the P5 promoter, including two within the P5 first exon itself. We used normal peripheral blood leukocyte DNA from three individuals (H1, H2 and H7); the P1 and P2 CpG islands of H1 and H2 have been analyzed previously, showing them to be differentially methylated (P1) and unmethylated (P2) [7]. Placenta genomic DNA, isolated from a single donor, was also tested as it is a tissue with high expression of *PLAGL1* [7], but it does not appear to utilize P5 for transcription (Fig 1A).

**Fig 4:**
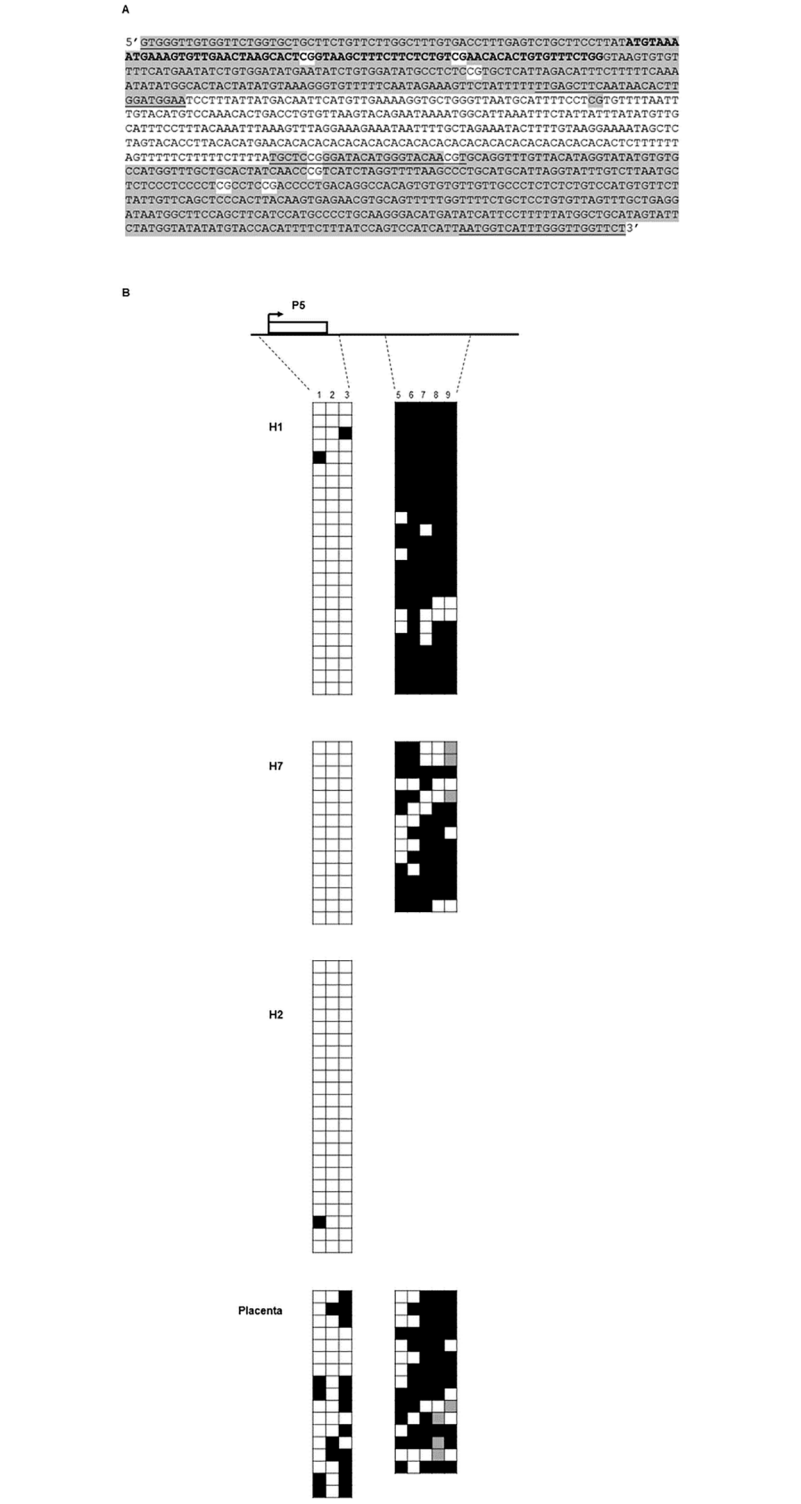
Methylation analysis of CpG sites close to the P5 TSS in peripheral blood leukocytes and placenta tissue. A) DNA sequence of the region close to the promoter P5 exon, written in the 5′-3′ orientation with respect to *PLAGL1* (chr6: 144054338–144055350; GRCh38/hg38). The 70-bp P5 exon is shown in bold. The amplicons for methylation analysis are highlighted in grey, and the locations of PCR primers are underlined. (Note: actual primer sequences are for bisulphite-converted DNA, see Materials and methods). Amplicon 1: CpG sites 1–3, labelled from 5′ end with respect to P5, are highlighted in white. CpG site 4 was not analyzed. Amplicon 2: CpG sites 5–9 highlighted in white (note that CpGs 5 and 6 are located within a primer). A further CpG in the UCSC Genome browser reference sequence is a SNP (rs6901529). B) Methylation analysis by bisulphite sequencing for the two separate amplicons for peripheral blood leukocyte genomic DNA (individual H1 and H7, and amplicon 1 only for H2) and placenta genomic DNA. Squares represent CpG sites: black indicates a methylated CpG site; white, an unmethylated CpG site. Grey shading indicates a CpG site that was neither CG nor TG.

The low CpG density necessitated sequencing of two amplicons to assess the methylation status of a P5 genomic region spanning ten CpG sites (Fig 4). Amplicons containing CpG sites 1–3 (region 1) and 5–9 (region 2) were studied (highlighted as grey sequence; Fig 4A). The fourth CpG site was not tested. A minimum of 14 plasmid colonies were sequenced, generated from the cloning of each amplicon. CpG sites 1–3, located within the P5 first exon, were unmethylated in all three leukocyte samples tested (Fig 4B), consistent with their location close to an active TSS. These CpGs were also predominantly unmethylated in placenta genomic DNA, a tissue that does not express P5 transcripts (Fig 4B). In contrast, CpG sites 5–9, located within the first intron, were predominantly methylated in sample H1, H7 and placenta (for sample H2, data were not obtained). We noticed that a small number of CpG sites in region 2 were no longer CpG dinucleotides (grey boxes); most were observed as TA.

Although some unmethylated CpG sites were observed in region 2, there was no evidence of differential methylation of alleles in leukocytes or placenta to suggest genomic imprinting, regardless of whether it was a P5-expressing or P5 non-expressing tissue.

### P5 transcripts escape genomic imprinting and are biallelically expressed

The lack of differential methylation suggested that P5 transcripts may be derived from both alleles. The allelic origin of P5 transcripts was determined using a single nucleotide polymorphism (SNP; rs2092894) located within an extended, alternatively-spliced version of exon 3 in the 5′-UTR of *PLAGL1*, as described previously [7]. The extended exon (which we have termed ‘exon 3b’) includes the 72-bp sequence ‘exon 3a’ but it is extended at the 3' end where the SNP is located (Fig 2). Therefore, the SNP is included within a subset of *PLAGL1* transcripts. We suspect that ‘exon 3b’ is a relatively large exon, as we have been unable to define the 3' end by RT-PCR using standard conditions.

In a heterozygous individual, rs2092894 is informative for all promoters due to its close proximity to the alternative first exons within *PLAGL1* transcripts. Using peripheral blood leukocyte cDNA from such a heterozygous individual (H3), RT-PCR amplification of the 5′-UTR of transcripts from each *PLAGL1* promoter was performed to assay for mono- or biallelic transcription. In the same way, we have demonstrated that P1 promoter transcripts are monoallelic and P2 transcripts are biallelic in peripheral blood leukocytes [7].

**Fig 5:**
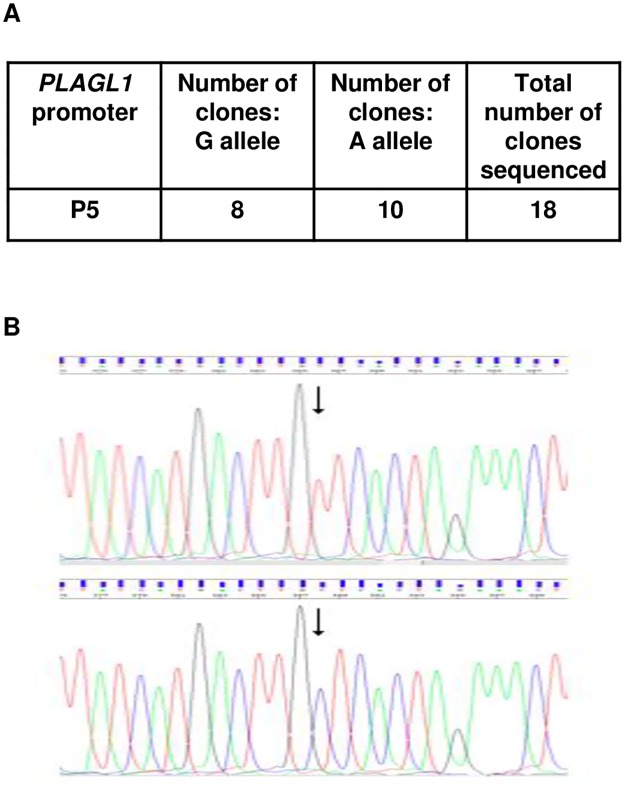
Allelic expression of *PLAGL1* transcripts derived from promoter P5, isolated from peripheral blood leukocytes from a normal individual (sample H3), heterozygous for SNP rs2092894. **A**: SNP alleles represented in cloned P5 transcripts indicate biallelic transcription as both alleles are represented. Data for P1 and P2 for sample H3 have been published previously, showing monoallelic and biallelic expression respectively [7]. **B:** Representative electropherograms of two cloned P5 transcripts indicating the alternative alleles at the location of the SNP (arrows). The sequence shown is 5'-3' with respect to the *PLAGL1* sequence. A representative sequence from this amplicon (clone 11; lower panel) has been deposited in GenBank (MF361142).

The resulting amplicons were cloned and individual clones were sequenced for each promoter. As anticipated, P5 transcripts were derived from both alleles, confirming biallelic expression from this promoter (Fig 5). Previously, P1 transcripts from the same individual were found to be monoallelic, with transcription only from the G allele (assumed to be the paternal allele), while P2 transcripts were biallelic [7].

### The P5 promoter is located within a MIR3 SINE retrotransposon

RepeatMasker analysis [13] (displayed in the UCSC genome browser) shows that the P5 promoter exon lies within a SINE retrotransposon of the MIR3 class. MIR3 repeats (Mammalian-wide Interspersed Repeat) are SINEs which exist only in mammals, and are the most ancient of transposable elements [14,15,16]. The SINE is 171-bp in length (chr6:144,055,219–144,055,389; GRCh38/hg38), and it spans the whole P5 exon as defined by 5' RLM-RACE, except for 2-bp at the 3′ end. Therefore, the P5 promoter appears to have evolved from the insertion of this retrotransposon within the *PLAGL1* locus.

**Fig 6:**
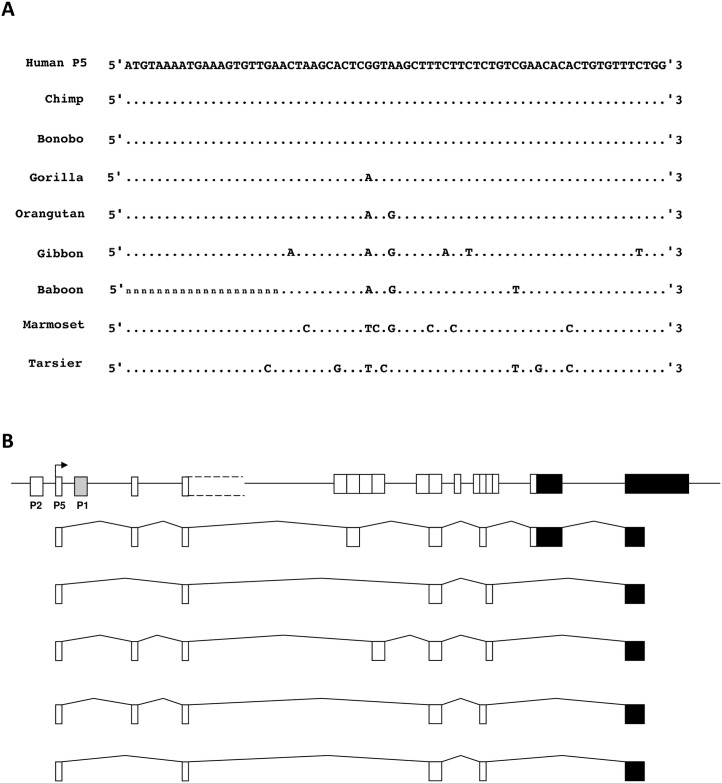
**Fig 6A: Comparison of the 70-bp P5 exon sequence between human and eight primate species.** Data is from the UCSC genome browser. A dot indicates a conserved nucleotide compared to the human sequence, and ‘n’ indicates where sequence is not available. **Fig 6B: Alternative splicing of transcripts derived from the *PLAGL1* P5 promoter, isolated from the *Pan troglodytes* lymphoblastoid cell line, EB176 (JC).** The *PLAGL1* locus is shown at the top (not to scale). Transcripts from P1 and P2 were also isolated from EB176 (JC) and sequenced (accession numbers in Materials and methods).

BLAT searches revealed high nucleotide sequence conservation of the P5 exon in primates. We detected an orthologous exon in the genomes of all primates available in the UCSC genome browser, although the sequence was partially missing in baboon, either due to a deletion or a gap in the published genomic sequence (Fig 6A). In chimp, this exon is also identified in the UCSC genome browser as being within a MIR3 SINE (88-bp, located at chr6:148,073,825–148,073,825; Chimp assembly Pan_tro 3.0/panTro5; May 2016). However, we could not detect a similar promoter exon in any non-primate genomes, including mouse and rat, suggesting that despite MIR3 being a mammalian-wide retrotransposon class, this promoter has evolved relatively recently, since the divergence of primates, and may have been subject to selective pressure for its retention [16]. Retrotransposons have been shown to contribute to the formation of new promoters, such as a novel, tissue-specific *KCNH5* promoter that is transcriptionally active in placenta [17]. Of all retrotransposons, MIR sequences have been found to be more frequently exonized within transcripts, possibly as a result of their ancient origin [16].

Little published data is available on *PLAGL1* expression in non-human primates, but it has been found to be maternally imprinted in the cynomolgus macaque (*Macaca fascicularis*), with evidence of biallelic expression in the liver [18]. For insight into the conservation of gene regulation, we cloned and sequenced transcripts from the P1, P2 and P5 promoters in chimpanzee (*Pan troglodytes*), using RNA extracted from the EB176(JC) lymphoblastoid cell line (Fig 6B; see Materials and methods for accession numbers). Transcripts from all three promoters were found to be expressed in chimp lymphoblastoid cells, and are highly similar to transcripts from the human orthologue. They are alternatively spliced, and encode either the long or short isoforms of the PLAGL1 protein, similar to human transcripts. The chimp P5 promoter region from this lymphoblastoid cell line also showed a similar methylation pattern to the P5 region in human leukocytes (S1 Fig), suggesting that P5 transcripts are biallelic in chimp. However, the EB176 (JC) cell line is homozygous at rs2092894 and suitable alternative SNPs could not be identified: therefore it was not possible to investigate genomic imprinting of transcripts from the *PLAGL1* promoters.

In conclusion, we have defined a novel, biallelically expressed *PLAGL1* promoter (P5) that is utilized predominantly in lymphocytes, particularly T cells. This provides further evidence to support our previous observations [7,9] that *PLAGL1* transcription escapes genomic imprinting in human blood cells. The P1, P2 and P5 promoter exons are conserved between humans and chimp and generate *PLAGL1* transcripts. A similar methylation pattern is observed at the P5 promoter region between humans and chimp, although we could not confirm conservation of monoallelic/biallelic expression due to a lack of informative SNPs.

As the P5 promoter exon appears to be present only in primates, it is likely that transcriptional control by *PLAGL/Plagl1* promoters in lymphocytes has diverged between humans and mice. Therefore it is important that the biallelic origin of transcripts in human blood cells (from the P2 and P5 promoters, rather than the imprinted P1, P3 and P4) is considered when mice are used as a model system for imprinting studies of this gene.

It has been proposed that imprinting at the *PLAGL1* locus is controlled by a 70-kb chromatin loop around the gene, regulated by CTCF-cohesin binding [6]. Our observations are consistent with this model, since the biallelic P2 and P5 promoters lie outside this domain. Despite this, it is possible that there is regulatory crosstalk between the P5 promoter and other *PLAGL1* promoters, for example, through transcriptional interference [19]. SINE retrotransposons can epigenetically reprogram gene promoters [20], and MIR retrotransposon sequences have been shown to function as insulators [21] or enhancers [16] within the human genome.

*PLAGL1* expression has been reported in CD4+ T cells from cord blood [22]. In mouse, *Plagl1* is active in T regulatory cells [23] and long term repopulating haematopoietic stem cells (LT-HSCs) [24]. Its expression has been found to distinguish between expression profiles in murine follicular helper cells (Tfh) and T helper 1 cells (Th1) cell populations [25]. As transcription is driven by P2 and P5 promoters in human blood cells, a more complex view of *PLAGL1* regulation and function is essential when considering loss of its expression in cells of haemopoietic origin and in haematological malignancies.

## Materials and methods

### RNA and DNA samples

A human tissue RNA panel (First Choice Human Total RNA Survey panel) and human peripheral blood leukocyte RNA were purchased from Ambion (ThermoFisher Scientific). Human blood cell RNA samples from CD4+, CD8+, CD14+ and CD19+ blood cell lineages were purchased from Yorkshire Bioscience, York, UK (http://york-bio.com/). Natural killer (NK) cell RNA (single donor) was kindly provided by Dr. Y. El-Sherbiny, School of Medicine, University of Leeds. All commercially-sourced RNA samples were derived from pooled cells from several donors. Genomic DNA samples were from peripheral blood leukocytes from three separate donors and placenta DNA was from a single, female donor (D3035; Sigma).

For Yorkshire Bioscience blood cell RNA samples, full details of blood cell isolation and *in vitro* activation prior to RNA extraction are available on the manufacturer’s website at http://york-bio.com/blood_cells_rna.htm. In brief, activated CD4+ cells were isolated after CD8+, CD19+ and partially CD14+-depleted mononuclear (MN) cells had been treated with 5mg/ml concanavalin A (ConA) for 3–4 days. Activated CD8+ cells were positively selected by immunomagnetic separation from CD14+ and CD4+-depleted MN cells following incubation with 5mg/ml phytohemagglutinin (PHA) for 3 days. Activated CD19+ cells were positively selected from CD14+ and CD8+-depleted MN cells following incubation with 2mg/ml pokeweed mitogen (PWM) for 4 days (Yorkshire Bioscience).

The chimpanzee lymphoblastoid cell line EB176(JC) was obtained from ECACC, and maintained in RPMI with glutamine and 15% foetal calf serum at 37°C, 5%CO_2_. RNA and DNA were prepared using Trizol (ThermoFisher) or the QIAamp DNA Mini kit (Qiagen), respectively.

### Reverse transcription PCR (RT-PCR)

First-strand cDNA synthesis was carried out using the ThermoScript^™^ RT-PCR system (ThermoFisher Scientific), with random hexamers according to the manufacturer’s instructions. For Fig 1A and 1B, three individual PCR reactions were performed for each tissue, using forward primers that specifically amplify transcripts from either the P1, P2 or P5 promoter, combined with a common reverse primer (located in the coding region in the final exon) in separate reactions. The same quantity of cDNA template was used in each PCR reaction. Forward primers: P1, dCTGAGCTCCGGGGGTCGT; P2, dGCTCCGGACTCCAGAACTT; P5, dCGGTAAGCTTTCTTCTCTGTCGAAC. Reverse primer: dACACTGGTGAGATTTCTGGGGAGAAT. PCR conditions were 95°C, 5 min; followed by 35 cycles of 95°C for 30 s; 60°C for 30 s; 72°C for 30 s; with a final extension step of 72°C for 3 min.

### Cloning and sequencing of transcripts

For sequencing, transcripts were ligated into pGEM^®^-T Easy vector (Promega), transformed into DH5α cells (Invitrogen) and plated on LB agar plates containing ampicillin, X-gal and IPTG (Sigma). Plasmid DNA was prepared from individual colonies using a GenElute Plasmid Miniprep kit (Sigma) and was sequenced using a BigDye^®^ v.3.1 Cycle Sequencing kit (ThermoFisher). Sequencing was carried out in both directions, and reactions were analysed on an Applied Biosystems ABI3130xl sequencer using Sequencing Analysis v5.2 software. Accession numbers: Fig 2A) activated CD4+ cell transcripts: MF084771; MF084772; MF084773; MF084774; MF084775; MF084776; MF084777; MF084778; MF084779; MF084780; Fig 2B) activated CD19+ cell transcripts: MF084781; MF084782; MF084783; MF084784; MF084785; MF084786; MF084787; Fig 6B) Chimp lymphoblastoid cell EB176(JC) transcripts generated from Promoter 5: MF361132; MF361133; MF361134; MF361135; MF361136 Additional chimp transcripts for Promoter 1; MF361137; MF361138; and Promoter 2: MF361139; MF361140; MF361141.

### Quantitative (Real-Time) PCR (qPCR)

QPCR was carried out using Taqman PCR reagents, according to the manufacturer’s instructions (Life Technologies, ThermoFisher Scientific). cDNA was generated from blood cell and tissue RNA, as described above. Reactions were performed on an ABI 7900HT Real-Time PCR system and analyzed using SDS v.2.3 software. Custom-made Taqman assays were used for the *PLAGL1* P2 or P5 promoters. The P5 assay was designed to specifically amplify transcripts containing the P5 first exon, the second and third exons of the 5'UTR. The probe was designed to cross the P5 exon-exon 2 boundary in transcripts. Experimental cDNA samples were run with either the P2 or P5 assay, in tandem with an assay to the endogenous control gene *RPLP0* (4333761F; Life Technologies), in separate reactions on the same plate. Expression of *RPLP0* was used to normalize *PLAGL1* expression for each sample.

The absolute quantification method was used for analysis: dilution series for standard curves were generated using CD4+ RNA (Yorkshire Bioscience) for the P5 analysis, and peripheral blood leukocyte RNA (Ambion) for the P2 analysis. Samples were run in triplicate with no template controls (NTC) included. NTC wells were negative in all cases. Details of the P2 Taqman assay (assay P2b) have been published previously [7]. Sequences for the P5 assay were as follows: Forward primer dGCTTTCTTCTCTGTCGAACACACT; reverse primer dGCAATCAAAAGCCAATCACGATGTT; probe 6FAM-dCAGGAACAATCCAGAAACA.

### 5′ RLM-RACE

For 5′-RACE experiments, the FirstChoice RLM-RACE kit was used (Ambion), according to the manufacturer's instructions, with activated CD4+ and activated CD19+ RNA (Yorkshire Bioscience). Nested reverse primers to exon 3a were used in two rounds of PCR: 1^st^ round: 5′ RACE Outer Primer (Ambion) and reverse primer dATGTGACACGAGGCAGCAG; 2^nd^ round 5′ RACE Inner Primer (Ambion) and reverse primer dGCCACATTAGACGTGACAGC. Ambion primers were designed to the ‘5′ RACEAdapter’ sequence at the 5′ end, and *PLAGL1* reverse primers were designed to exon ‘3a’.

### Methylation analysis by bisulphite sequencing

Bisulphite conversion of peripheral blood leukocyte genomic DNA was carried out using the EpiTect Bisulfite kit (Qiagen). Two adjacent genomic regions were PCR-amplified: amplicon 1 contained CpG sites 1–3 and amplicon 2 contained CpG sites 5–9. PCR primers were designed to amplify methylated and unmethylated sequences non-selectively. Primers were:

P5 Amplicon 1 Forward, dGTGGGTTGTGGTTTTGGTGT;

P5 Amplicon 1 Reverse, dTTCCATCCAAATATTATTAAAACTCAA;

P5 Amplicon 2 Forward, dTGTTTYGGGATATATGGGTATAAYGT;

P5 Amplicon 2 Reverse, dAAAACCAACCCAAATAACCATT.

The region around CpG site 4 was not analysed as suitable primers could not be designed. Bisulphite PCR was performed as follows: 94°C, 3 min; 35 cycles of 94°C for 30 s, 58°C for 30 s, 72°C for 30 s; with a final extension step of 72°C for 3 min. PCR products were cloned into DH10B cells (Invitrogen), sequenced and analysed as described previously [9]. Sequencing was carried out in both directions, and analysis was carried out using CpGviewer (http://dna.leeds.ac.uk/cpgviewer) [26]. For methylation of the chimp P5 promoter, the same procedure was carried out using DNA extracted from the EB176 (JC) cell line and the same primers as above.

### Analysis of allelic expression of P5 transcripts

For biallelic or monoallelic expression analysis of promoter P5, SNP rs2092894 was used (dbSNP, NCBI), as described previously for assessing P1 and P2 [7]. Peripheral blood leukocyte RNA was from a single, heterozygous donor (H3), and was reverse-transcribed as described above. For RT-PCR of *PLAGL1* transcripts, a forward primer specific for the P5 first exon was used, combined with a reverse primer to exon ‘3b’ (Fig 2): P5 forward, dCGGTAAGCTTTCTTCTCTGTCGAAC; reverse primer: dTGGTGGACCCTACCTCAGTT. PCR products were cloned and individual clones were sequenced, as described above, to determine the number of transcripts from each allele. Accession number for a representative transcript (see Fig 5B; clone 11): MF361142.

## Supporting information

S1 Fig Methylation analysis of the chimpanzee P5 promoter region using DNA from chimp lymphoblastoid cells. The same CpG sites were analysed as for the methylation analysis of the human P5 promoter region (Fig 4) using the same primers (see Materials and methods). A similar methylation pattern was observed compared to human leukocytes (Fig 4B).
(TIF)

## Abbreviations

bp: base pairs
CGI: CpG island
EST: expressed sequence tag
MIR: mammalian-wide interspersed repeat
PLAGL1: Pleomorphic adenoma gene-like 1
RLM-RACE: RNA ligase-mediated rapid amplification of cDNA ends
SINE: short interspersed nuclear element
SNP: single nucleotide polymorphism
TSS: transcriptional start site
UTR: untranslated region
