# Supplementary figures and images for: A tissue-specific promoter derived from a SINE retrotransposon drives biallelic expression of PLAGL1 in human lymphocytes
Source: PLoS One. 2017 Sep 28;12(9):e0185678. doi: 10.1371/journal.pone.0185678 (PMC5619815; doi:10.1371/journal.pone.0185678)

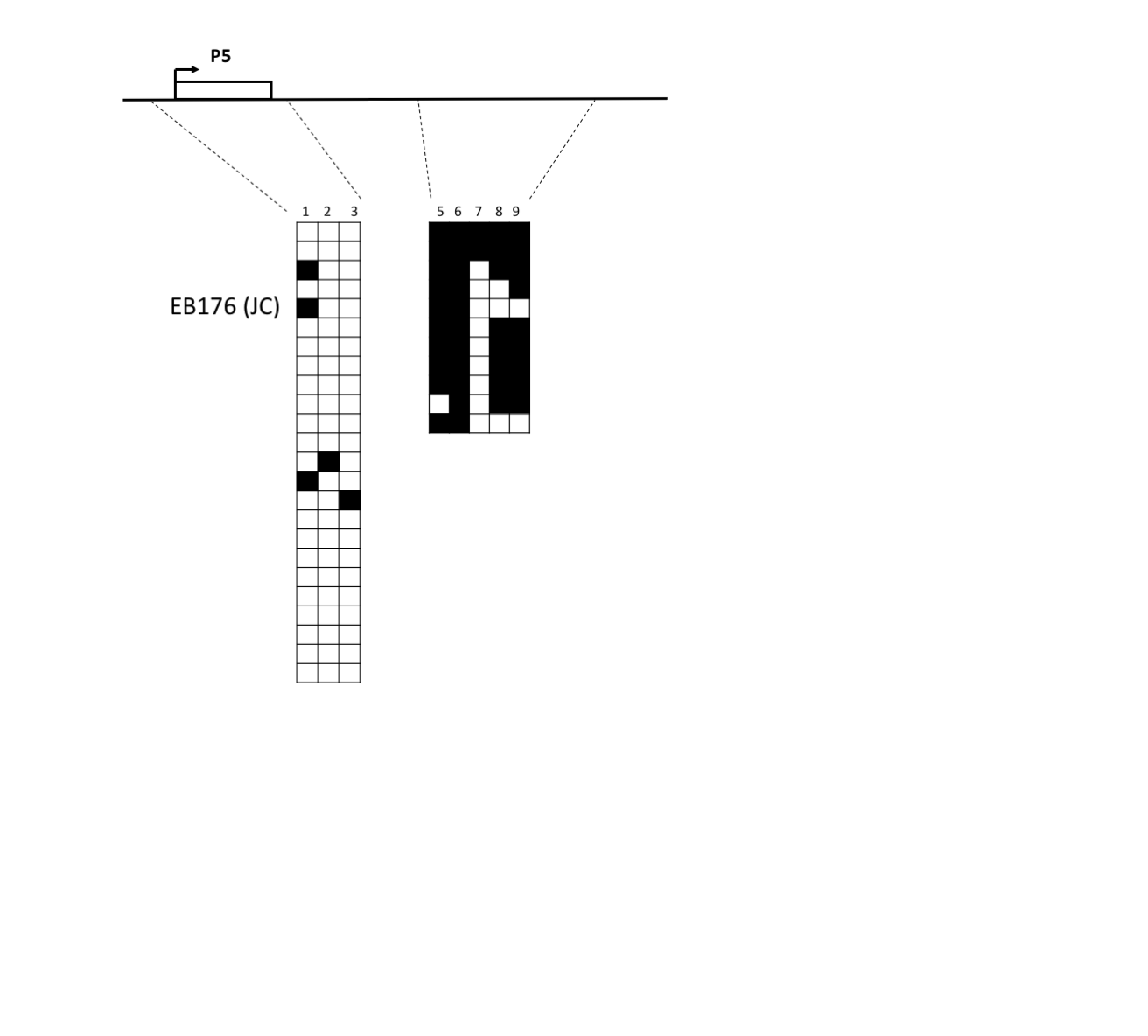

Supplement: S1 Fig — The same CpG sites were analysed as for the methylation analysis of the human P5 promoter region (Fig 4) using the same primers (see Materials and methods). A similar methylation pattern was observed compared to human leukocytes (Fig 4B). (TIF) [file pone.0185678.s001.tif]
